# A critical review on the welfare of Japanese quail in cage-free housing: Current knowledge and future perspectives

**DOI:** 10.1016/j.psj.2025.105263

**Published:** 2025-05-08

**Authors:** Désirée S. Jansson, Frida Lundmark Hedman, Jenny Yngvesson, Linda Keeling, Rie Henriksen

**Affiliations:** a Department of Clinical Sciences, Section for pig and poultry medicine, Swedish University of Agricultural Sciences, Box 7054, SE750 07, Uppsala, Sweden; b Department of Applied Animal Science and Welfare, Swedish University of Agricultural Sciences, P.O. Box 234, SE-532 23 Skara, Sweden; c Department of Applied Animal Science and Welfare, Swedish University of Agricultural Sciences, P.O. Box 7068, SE750 07 Uppsala, Sweden; d AVIAN Behavioural Physiology and Genomics Group, IFM Biology, Linköping University, 58183 Linköping, Sweden

**Keywords:** Japanese quail, cage-free housing, poultry production, animal welfare

## Abstract

The Japanese quail (*Coturnix japonica*) is an increasingly popular species in poultry production. Concern about poultry welfare, including quail, has resulted in a stronger focus for farms to transition to cage-free housing as highlighted by the 2020 European Citizens' Initiative ‘End the Cage Age’. Knowledge about how to design cage-free housing to accommodate Japanese quail’s behavioral and physiological needs is scarce, and there are currently no standardized regulations regarding this type of housing for quail in the EU. Based on available literature, we review current information on the specific needs and requirements of quail to facilitate the transition to cage-free systems. Overall, the literature shows that Japanese quail spend most of their time on the ground, often pecking, scratching, or hiding under cover, that they will lay eggs in nests if these are provided and appropriately designed, and that they dustbathe if given substrate to do so. However, information about optimal group size, stocking density, nest design, and appropriate litter substrates have not been sufficiently well researched in non-cage systems, and neither has the design of cage-free housing when large groups of quail are housed together. Additionally, guidelines regarding successful management of the reportedly high level of agonistic behavior performed by male quail housed in groups is missing, as well as instructions on the ideal sex ratio in large breeding flocks. In addition to controlled experimental studies to fill specific knowledge gaps in these areas, we suggest precision livestock farming technologies such as real-time image analysis and modelling using artificial intelligence to gather this information on farms and/or in research studies. We also suggest using genomic selection to tackle the high levels of agonistic behavior reported in male Japanese quail by identifying the genetic architecture underlying this trait to facilitate faster selection against it. While phasing out caged housing for Japanese quail has been suggested to ensure better animal welfare, this review highlights that more information and research are needed to guarantee that this transition doesn’t introduce new welfare and general managing problems in quail. We suggest that knowledge and experience about this transition from other poultry species, especially laying hens, can be used to facilitate the transition.

## Introduction

Japanese quail (*Coturnix japonica)* are farmed worldwide for both meat and egg production (Minvielle, 2004). The production is low compared to that of chickens, but quail are becoming increasingly popular as a source of protein both on large- and small-scale farms (Lukanov, 2019). They have been and continue to be a popular species for game farming in certain parts of the world (Caravaca et al., 2022) as well as being kept for research and hobby purposes (EFSA AHAW Panel, 2023A). Despite being the smallest farmed bird, an estimated 10 % of all table eggs in the world are produced by quail, and quail meat represents about 0.2 % of the global poultry meat production (Lukanov, 2019). The increase in quail production has mainly been attributed to their early sexual maturity, high egg production, and efficient feed conversion, as well as resistance to disease (Shanaway, 1994). Furthermore, their quick return over investment has also made them popular in middle- and low-income countries (Shalome and Nojuvwevwo, 2021). As their popularity as a farmed species continues to grow, so does concern regarding the provenance of quail products, including bird welfare.

**Fig. 1:**
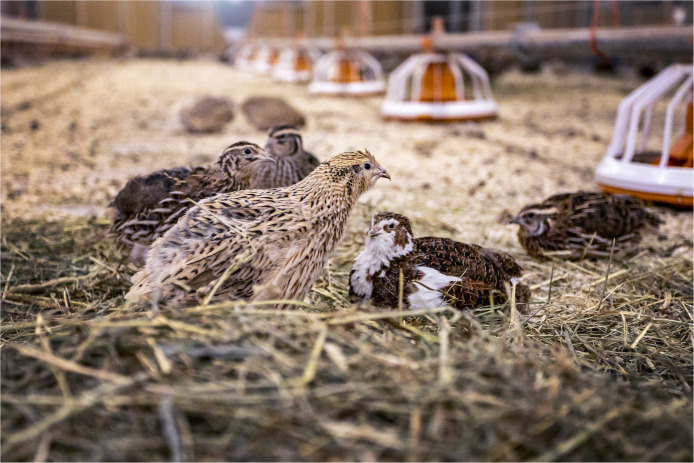
Japanese quail housed in cage-free system on sawdust (photo: Stjärnägg AB).

In today’s production systems, Japanese quail are farmed for eggs or meat or for dual purpose (eggs and meat) (Lukanov, 2019). While cage-free housing is common in certain countries where quail meat production is prevalent, layer, dual-purpose quail, and breeding quail are often housed in cages, albeit litter-based housing (see Fig. 1 of Japanese quail housed on sawdust in a non-cage system) or a combination of floor–cage housing also exists (Lukanov and Pavlova, 2020A,B; Lukanov et al., 2023). In such housing systems, a common practice is to brood and raise chicks on litter during their growth period and then transfer them to cages at the start of lay (Shanaway, 1994). Several welfare problems have been highlighted in intensive quail production, such as head injuries caused by aggressive pecking between males or as a consequence of escape responses, as well as reports on feather damage and foot lesions (reviewed by Gerken and Mills, 1993). Despite the increasing popularity of quail production and the Japanese quail’s role as a model species within genetics, behavioral, and developmental biology (Cheng et al., 2010), very little research attention has been given to the quail as a commercial bird compared to chickens (Minvielle, 2004). This might explain why there is no specific EU legislation for quail housing/management (EFSA AHAW Panel, 2023A), and current regulations by FAO only include recommendations regarding stocking density and do not take the behavioral and physiological needs of this species into consideration (FAO 2008, Ramankevich et al., 2022). However, there is a general agreement among welfare scientists that poultry benefit from non-cage housing since it promotes mobility and allows a wider spectrum and more frequent expression of the bird’s natural behavior (Hartcher and Jones, 2017).

Between 2018 and 2020, 1.4 million EU citizens signed the petition ‘End the cage age’, resulting in a commitment by the European Commission to put forward a legislative proposal by 2023 to phase out and finally prohibit the use of cages for poultry, including quail and some other farm animal species (www.endthecageage.eu). Based on this initiative and the recently published EFSA report on the welfare of ducks, geese, and quail on farms (EFSA AHAW Panel, 2023A), we take a critical look at the literature on cage-free housing of quail. The aim of this review is to establish what is currently known of Japanese quail needs and requirements in cage-free housing to facilitate a successful transition from cage to non-cage housing, as well as identify the knowledge gaps related to this species’ behavioral and physiological needs. Since the peer-reviewed literature on cage-free housing of quail is scarce, we also use the literature on cage-free housing of chickens (laying hens and broilers) as a resource for our review.

## Transitioning to non-cage housing

**Fig. 2:**
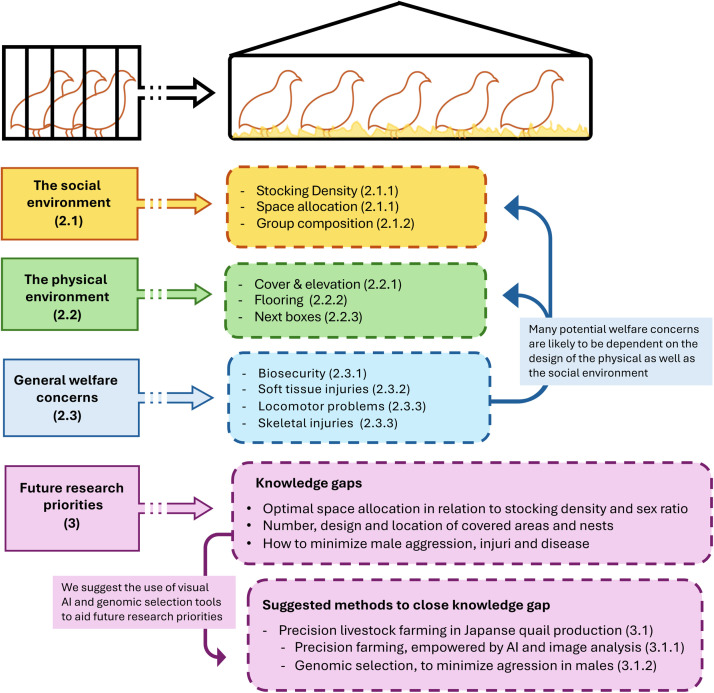
Flowchart showing the different focus areas covered in this review. References to relevant sections' numbers in the review are indicated in brackets.

From the experiences within the egg-laying poultry sector, we know that non-cage production systems come with a new set of challenges compared to caged systems that can compromise production as well as the welfare of the birds (Rodenburg et al., 2022; Blokhuis et al., 2007). These especially relate to the environmental conditions in barns and general management when large groups of birds are kept together, which can result in injuries, such as increased levels of feather pecking and keel bone damage in chickens (EFSA AHAW Panel, 2023AB, Relić et al., 2019). Microorganisms and parasites, especially those that are transmitted by the fecal-oral route, may be more difficult to control in non-caged housing, and with a transition from cage housing to cage-free housing, both the social environment and the physical environment will change for quail. They will be housed in larger groups in a larger enclosure, with more possibilities to express natural behavior and more conspecifics to interact with. Below, we discuss how best to design cage-free housing for quail, based on both group size and flock composition, as well as how to design the physical environment to better accommodate their behavioral and physical needs (see Fig. 2 for an overview of the different focus areas covered in this review). At the end of this section, we highlight injury and disease measurements that are often used to assess poultry welfare, and which can be applied to quail production to evaluate the success of the transition to cage-free systems.

### The social environment

#### Stocking density and space allocation

A natural consequence of housing quail in non-cage production systems is that a higher number of birds will be housed together as compared to in cages. In chickens (both broilers and laying hens), high stocking density in cage-free housing production systems has been shown to have a direct negative impact on the bird’s welfare by increasing social stress and competition for feed access as well as reducing the opportunity for birds to move around and thereby perform their natural behavior (Cetin et al., 2011, Stamp Dawkins et al., 2004, Simitzis et al., 2012). Nevertheless, information regarding optimal stocking density, group size, and overall space allocation for cage-free housing of quail is scarce, and only very few studies have explored the space requirements of quail in free-range settings.

Just like the chicken, Japanese quail is a social species with a dominance hierarchy based on a pecking order (Boag, 1982). In their natural habitat in East Asia, they spend most of their life in flocks, either in small breeding flocks or in larger flocks during the winter migration season (Chang et al., 2005; Lukanov and Pavlova, 2020A). Very few direct comparisons between stocking density in cages and non-cage housing exist in the quail literature, but those that do indicate reduced welfare and production of quail housed in cages compared to non-cage systems. When comparing the behavior and meat quality of caged (50×30×30 cm) quail, to small floor pen (60×60×30 cm) housed quail on sawdust, and quail housed on litter in larger enclosures (200×200×140 cm) with access to sand-bathing areas and nests, Muhammad and Mirza (2019) found that the latter expressed significantly less agonistic behavior and had better meat quality. Since both stocking density and enrichment differed between the three different housing conditions, it is difficult to distinguish between the effects of space allocation and stocking density from enrichment. Nonetheless, the study highlights how important space and enrichment are for the overall welfare and production quality of quail.

To date, studies on the effects of stocking density and space allocation in quail have mainly been done in cages with a space allocation in the range of 80 to 250 cm^2^/bird (El Sabry et al., 2022) and predominantly focused on physiological measurements of economic interest, such as growth and egg production. Most of these studies have reported that increased stocking density leads to reduced body weight gain, egg production, and feed conversion rate as well as increased mortality rate (Aro et al., 2021; El-Tarabany, 2016; Nagarajan et al., 1991; Wilson et al., 1978). Whether these correlations are due to increased social stress, stress from not being able to perform natural behavior, increased feed competition or disease burden, or a combination thereof is not clear since potential behavioral alterations due to reduced space allocation have largely been overlooked (reviewed by El Sabry et al., 2021). One of the few studies that looked at the benefits of decreasing stocking density in floor housing (Wilson, 1978), did this in bobwhite quail (*Colinus virginianus*) (a quail species of similar size to the Japanese quail) and concluded that when comparing stocking density variation between 113 and 929 cm^2^/bird, stocking density below 232 cm^2^/bird was associated with increased mortality and the authors recommended a minimum stocking density of 372 cm^2^/bird as a trade-off for production traits such as feed conversion ratio. This is a considerably lower stocking density and higher space allocation per quail than what is currently recommended by FAO (2008), where recommendations range between 180 and 200 cm^2^/adult quail and 20–25 birds per cage measuring 100×45×27 cm (FAO 2008, no recommendation exists for non-cage housing). Optimal total space allocation in a commercial farming situation and, thereby, also the most favorable minimum and maximum flock size seems not to have been explored in cage-free housing systems for quail. In cage-free systems for laying hens, several thousands of hens are often housed together, but even within these systems very few studies on the use of the range area have been carried out on larger (commercial-sized) flocks (exceptions include Appleby and Hughes, 1991, Grigor, 1993, Bubier and Bradshaw, 1998, Hegelund et al., 2005). Commercial quail flock sizes range from a few hundred individuals to several thousand (EFSA AHAW Panel, 2023A). Given the limited guidance on stocking density, which mainly seems to refer to cage-housed quail, we can assume that stocking density might also vary considerably between commercial farms. There should, therefore, be plenty of opportunity to use commercial quail farms to investigate the effects of space allocation and stocking density on quail behavior, physiology, and overall production outcome to gain better insight into the optimal space allocation in cage-free production systems. At the end of this review, we discuss how new technology might aid in providing these measurements.

#### Group composition

Studies have highlighted the occurrence of severe agonistic interactions when quail are housed together (Mills et al., 1997). In cage production, aggressive pecking has been reported to cause serious, sometimes lethal injuries such as skin lesions or eye loss (Mills et al., 1997). However, agonistic interactions can also occur when small groups of quail are kept at low stocking density (8 to 9 birds per 19 m^2^) in large semi-natural outdoor aviaries (Schmid and Wechsler, 1997). Injuries caused by aggressive pecking are mainly observed as a consequence of male aggression towards other males, although sexual aggression performed by males towards females, especially during mating, has also been reported (Pellegrini et al., 2019).

The occurrence of aggressive behavior towards conspecifics is low before the quail reach sexual maturity and rare in groups without adult males, indicating that this kind of agonistic behavior is linked to natural changes in the males’ behavior as they reach their reproductive stage (Wechsler and Schmid, 1998). Injuries caused by aggressive behavior are therefore mainly observed in breeding flocks when adult quail are kept in mixed-sex groups to obtain fertile eggs and not in laying flocks or in the meat-producing flock where quail are slaughtered before or around sexual maturity (Shanaway, 1994).

From the chicken literature, we know that aggressive pecking is distinct from feather pecking by having a different underlying motivation, i.e. establishing hierarchy, and is directed to the head and comb area rather than to the bird’s back as often occurs during feather pecking (Rodenburg et al., 2013; Buitenhuis and Kjaer, 2008). Although head injuries caused by aggressive pecking by males have been highlighted as an important welfare problem in quail farming (Mills et al., 1997), males will also grab, mount, and force cloacal contact onto other males as well as females. This kind of behavior is associated with strong aggressive pecks when performed between males (Caliva et al., 2017) and has therefore nearly always been proposed as aggression motivated to establish dominance (Schlinger and Callard, 1990). However, when this behavior is performed between males, it leads to a reduction in subsequent mating and fertilization success in the males when subsequently placed with a female. The reduction in mating and fertilization success is to a similar extent to a prior mating with a different female (Adkins-Regan, 2014), and it has therefore been suggested that a strong mating motivation could underlie these agonistic interactions (Adkins-Regan, 2014). The sexual and aggressive behavior of wild Japanese quail has not been reported, and a full understanding of the motivation underlying this kind of agonistic behavior is therefore missing.

The provision of visual barriers and the age of introduction do not seem to influence the rate of aggressive pecking between quail, and neither does light intensity (15 lux versus 170 lux) (Wechsler and Schmid, 1998). Only reducing the light intensity to almost complete darkness (1 to 5 lux) resulted in a significant decrease in pecking rates, but even under this condition, some males had to be removed because of serious head injuries (Wechsler and Schmid, 1998).

One solution (besides beak trimming and anti-pecking devices) to reduce the occurrence of injuries caused by aggressive pecking between quail that has been put forward is to lower the sex ratio between males and females in breeding flocks. Several studies have measured fertility in groups of quail with different male-to-female sex ratios, in the range of 1:6 to 1:20 in single-male groups (Wechsler and Schmid, 1998; Woodard and Abplanalp, 1967; Narahari et al., 1988). Egg fertility was as high as 86 –92 % in groups with 6 –8 hens per male, and only in groups with 20 females per male was the percentage of fertilized eggs significantly lower (69 %). Reducing the sex ratio could, therefore, be a way of reducing potential male aggression in breeding flocks without negatively affecting fertility, although the occurrence of male aggression in relation to the sex ratio still needs to be established under field conditions.

From studies on domestic turkeys and chickens, we know that up to a certain group size, birds are able to distinguish between familiar and unfamiliar conspecifics, which affects the level of aggression (Buchwalder and Huber-Eicher, 2005; D’Eath and Keeling, 2003). In turkeys, the level of aggression toward unfamiliar and newly introduced males to an established flock is negatively correlated with group size and allocated space, suggesting that allocating more space to a flock and keeping birds in larger flocks could potentially reduce agonistic interactions between males (Buchwalder and Huber-Eicher, 2005). To date, no studies have investigated the level of aggression between quail when kept in groups of 100–1000s, and most studies on agonistic behavior in quail have been done on groups of up to 30 individuals (Wechsler and Schmid, 1998). Future studies on quail agonistic behavior in commercial settings could, therefore, benefit from taking group size into consideration.

Quail aggressiveness has been shown to vary substantially between individual males (Guzman et al., 2013; Pellegrini, 2019), meaning that some males are consistently more aggressive than others. Studies have indicated that genetic differences form the basis of much of the variation in aggressiveness between quail (Maekawa et al., 2018; Recoquillay et al., 2015). This suggests that identification of individuals with an aggressive profile could be a key step to introduce management practices aimed at minimizing aggression within a flock (Jones, 1996; Jones and Hocking, 1999), for example, via genetic precision farming as discussed at the end of this review.

Due to the low reported level of agonistic interactions between females, we would not expect severe problems with aggressive behavior in all-female groups for table egg production and in groups composed of several females and one male only for brood egg production. These types of groups might, therefore, be kept successfully both in large and small flocks, although breeding flocks over a certain size might require more males in the flock to sustain high egg fertility. Social instability in female-only housing environments seems to increase the occurrence of agonistic behavior in female Japanese quail (Guibert et al., 2010), demonstrating that agonistic behavior between females does exist and that the social environment is still important for the overall welfare of female quail. Furthermore, this kind of social instability might not just affect the females negatively but can also affect their offspring. One study found that quail chicks hatched from eggs laid by females housed in an unstable group hatched later, developed more slowly post-hatch, and were more anxious than those hatched from eggs laid by females in stable groups (Guibert et al., 2010). This means that a suboptimal social environment not only negatively affects the welfare of quail housed in that environment but also affects the welfare of their offspring, which will be important to take into consideration when housing breeding flocks (Charrier et al., 2022).

### The physical environment

The possibility to express natural behavior is now an accepted dimension of the definition of good animal welfare, and a mismatch between these behavioral needs of poultry and the environment they are housed in often leads to welfare problems (Bracke and Hopster, 2006; Buller et al., 2020; Dawkins, 2023). Below, we look at the behavioral repertoire of quail in nature and compare it to the few studies that have quantified the behavior of quail in non-cage housing to determine which behaviors they are strongly motivated to perform and what physical resources are needed in their housing environment for them to be able to express these.

#### Cover and elevation

The wild Japanese quail is a migratory bird with an estimated migration distance of 400–1000 km (Wakasugi, 1984). During the breeding season, however, only short flights are observed as an anti-predator response (Taka-Tsukasa, 1935). Although domesticated quail seem to have lost their propensity to migrate (Derégnaucourt et al., 2005), they are still able to fly as a secondary defense mechanism. If they can't run away for cover, they will throw themselves up into the air to escape (Cheng et al., 2010). This vertical escape mechanism in quail can lead to serious traumatic head injuries if the roof is at an inappropriate height (Cheng et al., 2010).

Unlike chickens, Japanese quail are not motivated to perch or use elevated structures to feel safe from potential predators, especially at night (Schmid and Wechsler, 1997). Instead, quail seem to be highly motivated to seek out cover at ground level. If given access to cover on the floor, quail prefer to spend a large part of their time there rather than in open areas of the enclosure (Schmid and Wechsler, 1997). In one study, domesticated quail sought cover almost half (48 %) of the time, even though only 17 % of the floor surface was covered (Schmid and Wechsler, 1997). If given access to cover, Japanese quail show fewer escape behaviors associated with stress than if cover was absent (Buchwalder et al., 1997). In the wild, Japanese quail are usually found in dense vegetation, and studies on quail kept in captivity have shown that they seem to prefer cover that is completely or partially open to the sides over those that are partially open from the top (Buchwalder et al., 1997).

#### Flooring

Studies quantifying the behavior of quail in semi-natural aviaries with *ad libitum* access to feed have found that quail spent 24 % of the observation time walking and running and 8 % pecking and scratching at the ground (Schmid and Wechsler, 1997). In their natural habitat, Japanese quail feed on grass seeds, peas, grains, berries, young shoots, tender leaves, insects, and other small grubs (Taka-Tsukasa, 1935). As these feed sources are likely to be dispersed, foraging activity probably accounts for a large proportion of the daily activity in wild quail. Earlier studies have also reported that quail spent around 10 % of their time preening and 4 % dustbathing (Statkiewicz and Schein, 1980). The quail showed high levels of dustbathing when the deprivation of a dustbathing substrate was terminated, and they performed vacuum dustbathing when kept in cages without a dustbathing substrate (Gerken and Mills, 1993). The large litter area in non-cage systems allows for foraging and dust bathing behavior, which are both important for welfare in poultry, including quail, but also result in much higher dust levels in the housing environment compared with cages (Rodenburg et al., 2005). Therefore, a potential major disadvantage of non-cage systems that needs to be accounted for is the high dust levels compared with caged housing.

As highlighted in the chicken literature, birds in floor systems have increased contact with their feces, which especially promotes the transmission of pathogens that are transmitted by the fecal-oral route. Also, the equipment in these systems may provide more hiding places for the red poultry mite (*Dermanyssus gallinae),* and poultry roundworms such as *Ascaridia galli* and *Heterakis gallinarum* are more prevalent in non-cage systems for chickens (Jansson et al., 2010; Thapa et al., 2015). All this can present increased risks for infectious diseases such as coccidiosis (*Eimeria* spp.) and colibacillosis (*E. coli* infection) (Rodenburg et al., 2020). Since quail spend most of their time on the litter, and unlike chickens, never roost, the environmental conditions within the barn might be even more important. Maintaining good hygiene and applying appropriate cleaning routines are therefore of utmost importance, as well as choosing the best litter material (Monira et al., 2003). Litter serves several functions that include thermal insulation, moisture absorption, and a protective barrier from the ground. Its quality is considered a crucial factor for poultry welfare (Savory, 1995) since it plays an important role in leg and skin health as well as permits birds' natural scratching behavior (Škrbić et al., 2012; Arnould et al., 2004). There is currently little information on the behavior and overall health condition of quail kept on different litter materials under commercial conditions. Different litter types (sand, dried mud, sawdust, wheat straw, and rice straw) have been suggested for cage-free housing of quail based on the chicken literature, but it has hardly been investigated for use in quail. Mohammed and colleagues (2017) tested day-old quail chicks on different litter types and reported that maintenance behaviors (eating, crouching, huddling, sitting, and preening) were more frequent on sawdust, and birds reared on sawdust also had the lowest frequencies of feather pecking and better growth performance. While studies exploring the condition of adult quail kept on different types of litter in cage-free systems are currently lacking, the preference of different litter substrates, their effect on overall animal welfare as well as ease of management including cleaning and costs have been studied in chickens (Monckton et al., 2020; Jansson et al., 2010). The quail industry can therefore use a lot of the information that has already been acquired from broilers and laying hens kept in loose-house systems about optimal litter substrate, which should facilitate the transition of quail to cage-free housing systems.

#### Nest boxes

Just like chickens, quail prefer to lay eggs in nest boxes as opposed to laying them out in the open (Schmid and Wechsler, 1998). In both egg production and research, Japanese quail are often housed in cages without nest boxes, and in such conditions, quail hens display pre-laying restlessness (Schmid and Wechsler, 1998). The inability to perform normal pre-laying behavior is regarded as a highly important problem for the welfare of caged laying hens (Weeks and Nicol, 2006) and seems to be of equal importance to quail females.

When given the option to choose between different types of nests, Schmid and Wechsler (1998) observed that female quail preferred to lay eggs under cover with a small opening but not in nests with multiple openings. This suggests that they prefer to be concealed as much as possible during egg laying, which is also supported by the fact that the percentage of eggs laid outside the nest areas on the floor was significantly lower in pens with a high light intensity (170 lux) compared with low (15 lux) light intensity (Schmid and Wechsler, 1998). Michel (1989) reported that quail preferred nest boxes with vertical dark and light stripes over identical nest boxes with horizontal stripes or uniform grey sides and green nests over yellow-red ones. This indicates that quail have specific preferences concerning the design of their nesting site, perhaps preferring camouflaged nest boxes, although more research is needed to support this hypothesis. If quail are provided with nest boxes close to the walls in their housing system and dustbathing boxes more centrally placed, they lay up to 90 % of their eggs in nest boxes (Schmid and Wechsler, 1998). Furthermore, nest boxes situated in the corners of the pens seem to be preferentially selected for egg laying as opposed to centrally placed nests (Schmid and Wechsler, 1998). Nest design and location therefore both seem to play an important role for motivating quail to use the nest. This knowledge could be especially important for designing a proper cage-free system that allows for fast and secure egg collection by motivating the birds to lay eggs only in specific areas of the pen. Yet some questions remain, such as flooring in the nests and how many nest boxes to install in relation to group size. Previous studies have found that quail prefer to lay eggs on litter over on perforated plastic (Schmid and Wechsler, 1998), but no studies have yet explored how many quail are willing to share nest boxes, and therefore, the optimal ratio of females to nest box is unknown.

### General welfare concerns

As mentioned above, transitioning from cage systems to non-cage systems is not without challenges that can compromise animal health and welfare. Different animal-based measures for welfare assessment have therefore been highlighted in chickens and other poultry species to assess and ensure a successful transition to loose-housing systems. Various welfare concerns linked to cage-free housing have thereby been highlighted. Research on animal health and welfare indicators in quail has received limited attention compared to chickens and turkeys. The spectrum of diseases and injuries in quail is similar to other gallinaceous birds, such as laying hens and broiler chickens. For this reason, some conclusions may be based on the literature from other poultry categories.

#### Biosecurity measures

Since cage-free housing may increase the risk of infection, poultry operations, including quail farms, need to rely on the implementation of biosecurity routines to preserve animal welfare and health and to reduce the need for antimicrobial treatment (WOAH, 2022). This has become even more important in recent years since the appearance and spread of avian influenza viruses (AIV). Like other gallinaceous birds, quail are highly susceptible to avian influenza, and it has been suggested that they may contribute to the evolution of AIV in Asia (Nguyen et al., 2016). Disease prevention through biosecurity is especially important when new housing and management routines are introduced or amended, and for this reason, biosecurity needs to be considered with some care when transitioning from caged to litter-based housing. Biosecurity encompasses a wide range of preventive measures applied to mitigate risks of introduction and transmission of infectious diseases at farm level, throughout a region or country, or even globally. It includes external (between farms) and internal (within farms) measures. It is easier to apply biosecurity measures when flocks are contained indoors, but they should also be applied in free-range flocks, irrespective of their size. Among the most important measures are restricted contact with wild birds and rodents, the use of dedicated tools and equipment, footwear and clothing for each flock, and strict visitor control and hygiene barriers when gaining access to poultry enclosures. Mixing of birds from different sources and age groups should be strictly avoided. Also, cleaning and disinfection during downtime between flocks is imperative to avoid transmission from residual pathogens from earlier flocks.

#### Soft tissue injuries

Soft-tissue injuries in quail range from hemorrhages, superficial skin damage, scratches, wounds, and skin/subcutaneous infections. Lesions may be caused by pecking and scratching by claws of other birds in the flock, by self-mutilation, rough handling, or from equipment and poor litter quality.

##### Injuries from fear/flight reactions

The typical vertical take-off panic/fear flight reaction in Japanese quail may cause severe head and neck injuries, sometimes with fatal outcomes. Such injuries may be prevented by appropriate housing design and enrichment. This type of injury is most common in cages and is prevented by low cage height, which limits the birds’ ability to gain upward-directed speed and force. It should be less of a problem in pens/aviaries as long as the ceiling is high enough to allow a full flight reaction, and the provision of cover will lessen the flight-inducing stimulus (Buchwalder and Wechsel, 1997).

##### Injurious aggressive pecking

In poultry, injurious pecking is considered to be associated with chronic stress and misdirected foraging and exploration behavior. In Japanese quail, aggressive pecking is predominantly a problem among mature males as these display a higher level of aggression compared with females. Aggressive pecking in Japanese quail may result in loss of feathers and skin/soft tissue lesions, especially on the head and eyes, and is a potentially serious welfare problem (Gerken and Mills, 1993). This behavior has been reported from small and large multi-male flocks alike, and under both intensive husbandry systems and from semi-natural outdoor aviaries (Schmid and Wechsler, 1997). Based on a series of experiments, Wechsler and Schmid (1998) recommended that quail breeding groups should be kept small and consist of a single male and 8-12 females, as none of the studied housing conditions appeared to reduce aggression. Moreover, sexual harassment of females by conspecific males and mating injuries may also occur in Japanese quail (Persaud et al., 2004).

##### Feather pecking

In chickens, the main reason for plumage damage is believed to be feather pecking, which is a form of injurious behavior where birds peck at, pull, and potentially remove and consume each other's feathers, resulting in feather damage (Savory, 1995; Bilčík and Keeling, 1999). It affects both males and females. Although it can occur in any housing system, this behavioral problem can spread more easily and become even more serious in cage-free flocks, where the larger group size means that more birds might develop the behavior or fall victim to it (Rodenburg et al., 2013). In quail, only a couple of studies have quantified feather pecking, and these found an overall low incidence (Miller et al., 2006; Nordi et al., 2012). It is, therefore, currently difficult to assess if feather pecking is as prevalent in quail production as in the chicken laying hen industry and if its occurrence is dependent on the housing systems. A more recent study showed that rearing quail on sawdust was associated with lower levels of feather pecking than other bedding materials (Mohammed et al., 2017).

##### Scratches and cellulitis

Cellulitis is a common sequel to skin lesions, particularly scratches from claws, in broiler chickens. Typically, cellulitis is detected during meat inspection at the slaughter plant, where discoloration of the skin and subcutaneous infections are observed. Cellulitis associated with the bacterium *Escherichia coli* (coliform cellulitis) and high rejection in meat-type Japanese quail has been reported (Burns et al., 2003), but the overall occurrence has not been reported.

##### Foot-pad dermatitis

It is well established that good litter condition is essential to prevent foot-pad dermatitis in poultry, and food-pad scoring is considered an important animal-based welfare indicator in broiler chickens (Welfare Quality®, 2009). Very limited information is available on the occurrence of foot-pad lesions on commercial quail farms but results from other poultry species can most likely be applied to quail.

#### Locomotory problems and skeletal injuries

In contrast to other poultry species, such as chickens and turkeys, locomotory problems in quail have received very limited attention in the literature. Moderate to severe lameness was reported at an average rate of 3.62 % and 1.23 %, respectively, in flocks of meat-type quail reared in indoor floor systems, but the causes were not described (EFSA AHAW Panel, 2023A). In an earlier report, Gerken and Mills (1993) described articular and peri‑articular bacterial infections in breeders and meat-type quail. Lameness in poultry may result from a variety of causes, including trauma (fractures and dislocations), infection, and developmental problems.

##### Keel bone damage

One of the main problems of cage-free housing in laying hens is the high occurrence of keel bone injuries (Riber et al., 2018). Keel bone fractures are currently considered to be one of the most serious welfare problems in commercial non-cage egg production because of its high prevalence, the pain involved during the weeks required to heal a fracture, and the reduced mobility during this time (Riber et al., 2018). These kinds of fractures have been reported to affect 30 to 95 % of the individuals in laying hen flocks housed in non-cage systems while in furnished cages these numbers are reported to be lower (between 15 and 55 %) (Hardin et al., 2019) It is believed that selection for early sexual maturity in laying hens and a continuous high egg production have led to increased bone fragility and susceptibility to fractures due to the high calcium requirement for the formation of eggshells (Riber et al., 2018). Additionally, collision with housing structures when flying and flapping combined with the weakened bone strength is considered the major risk factors for keel bone fractures in laying hens (Fleming et al., 2004) and might explain the higher occurrence of keel bone fracture in cage-free housing systems where the birds have space to move more freely.

Japanese quail have also been selected for continuous high egg production and have an even higher egg-to-body mass production ratio than chickens (Priti and Satish, 2014). Their high egg production combined with their capability of short flights within enclosures suggest that quail, like laying hens, might also be prone to keel bone damage, especially in cage-free housing. However, information on keel bone damage is almost non-existent in quail, although personal communication reported in the recent EFSA report on farm-housed quail (EFSA AHAW Panel, 2023A) indicates that the incidence of keel bone damage is around 0.5 % of cage-housed quail and 2.6 % of floor-housed birds. These numbers were supported in a recent evaluation of keel bone damage in laying quail, where the occurrence of keel bone fracture was reported to be 1.7 % in small cage-free housing (Hildebrand et al., 2024).

## Future research priorities for cage-free quail housing

There are currently no set guidelines in the EU that define what cage-free housing for Japanese quail should look like (EFSA AHAW Panel, 2023A), and therefore also no information on how to transition from cages to cage-free systems in a sustainable way that ensures a high level of animal welfare as well as being cost-effective for farmers. Based on the scientific literature, quail spends a substantial amount of time scratching and pecking at the ground if given enough space and substrate that allows for these behaviors. They are also likely to hide under cover, use nest boxes for egg laying, and dust bath substrates if these are provided. Although providing cover seems to have a positive effect on quail welfare, as demonstrated by fewer escape attempts and generally lower levels of anxiety, studies investigating the optimal size and location of cover within cage-free housing systems are currently lacking. Similarly, no studies have investigated the ideal number and location of nest boxes within non-cage systems. Cage-free multi-tier systems for laying hens have been developed to enable commercial farmers to maximize the use of available space in a cage-free system without compromising the minimum welfare needs of the hens. Given that quail don’t seem to roost or use elevated structures, such systems have not yet been considered for quail. However, considering that quail will spend a large amount of their time under cover, slightly elevated structures might be used by quail if these are covered and accessible by ramps. Such solutions would potentially increase the use of available space in cage-free systems without necessarily increasing the floor area of the barn. The optimal design of cage-free housing, including cover and nest area, is most likely dependent on the intended group size and the stocking density. As mentioned above, hardly any studies have investigated optimal group size and stocking density in cage-free housing systems for quails, but it is important to study in the future both from the perspective of the quails’ needs as well as functionality in a production setting.

More research is needed to transpose the information outlined here into alternative cage-free housing systems for Japanese quail. As we have seen from the chicken industry, it is not always easy to implement scientific findings on commercial farms, and this can delay a successful transition from cage to non-cage systems. However, the quail industry is not starting from scratch. A lot of knowledge from chickens and other galliform birds on how to assess and prevent several potential welfare problems in cage-free housing exists and can, therefore, be applied to quail. Especially regarding biosecurity and potential behavioral problems as well as injuries that are likely to be similar in quail. At the same time, the Japanese quail is a distinct species with its own unique behavioral ecology and, therefore, behavioral needs that need to be taken into consideration when designing cage-free housing. Quail are, for example, highly motivated to stay on the ground under cover, and the males, especially, are likely to perform highly agonistic behavior to other males and even females. Group size and stocking density, as well as sex ratio in breeding groups, are likely to influence the behavior of the quail, both regarding overall use of the housing systems and therefore the most optimal design but also concerning the degree of agonistic interaction between quail. A specific focus on how quail housed in large groups use cover and space is needed to best design the most optimal cage-free housing systems, as well as a specific focus on how to minimize the occurrence of agonistic behavior. To date, knowledge regarding the effect of stocking density and group size on agonistic behavior in quail is lacking, but the literature indicates that aggressive interactions are likely to be a major problem in groups with many adult males. While several studies have tried to modify the environment to prevent agonistic behavior (often unsuccessfully), none have tried to minimize their need to perform this behavior. Below, we discuss how genetic precision farming might be a valuable tool to select against highly agonistic behavior in quail, as well as how the use of technological precision farming tools could aid in the faster development of optimal cage-free systems for Japanese quail.

### Precision farming in Japanese quail production

Precision livestock farming is an advanced farming method that utilizes all available resources and technologies to improve process efficiency and optimize animal welfare (Banhazi et al., 2012). It is concerned with solving issues and problems related to productivity, sustainability, and animal health and well-being by providing a farm management system for real-time animal monitoring, environmental control, and decision support (Banhazi et al., 2012). With the recent advancement in artificial intelligence (AI) (Garcia et al., 2020), precision farming technologies are becoming more advanced and now provide non-invasive and accurate ways to monitor many animals independently on farms, with the ability to obtain a myriad of information, ranging from the animals’ use of the enclosure, disease and injury detection as well as general information related to productivity.

#### Precision farming, empowered by image analysis

As more quail on farms are housed in large groups of 100 s or 1000s of individuals, monitoring the health and behavior of all individuals daily is becoming increasingly difficult. At the core of animal welfare is a concern for the individual’s suffering, and information about the behavior of all birds is paramount when evaluating or developing the most optimal cage-free housing system. The use of deep learning in the context of animal welfare is a new interdisciplinary area of research in which modern methods from the field of AI are used to create new opportunities for assessing and evaluating animal welfare. This is a promising new avenue where, for example, a camera-based system using artificial intelligence is used for automated detection of behaviors and/or injuries in poultry or other farm animals. To date, computer vision and deep learning have been implemented in livestock farming research for different applications such as body weight estimation in cattle and pigs (Dohmen et al., 2022), disease detection in chickens (Mbelwa et al., 2021), behavior and posture recognition in broilers (Fang et al., 2021), carcass weight evaluation in ducks (Chen et al., 2023) and egg examination in chickens (Çevik et al., 2022). This kind of technology has also been used on quail, specifically to detect early embryonic development using thermal micro cameras (Nakaguchi and Ahamed, 2022) as well as to detect adult quail’s location on farms (Evangelista et al., 2022). The technology is under continuous development and is becoming more user-friendly as well as more precise in its measurements when tracking animals. Computer vision- and deep learning-based monitoring systems have the potential to provide an up-to-date evaluation of the welfare and health condition of large flocks of quail in real-time at the individual level, without having to rely on human on-site evaluation. The technology therefore has the potential to be a cost-effective tool for farmers to quickly assess disease outbreak on farms as well as obtain continuous information related to overall production efficiency. Additionally, it can also be used for research purposes to evaluate the usefulness of different housing designs and enrichment resources. This technology could, therefore, be a promising tool for future research aimed at investigating the effect of different cage-free housing designs, both regarding flock composition and size on the quail’s behavior and productivity as well as evaluating the quail use of different designs of the physical environment.

#### Genomic selection

To date, attempts to minimize or eliminate the occurrence of aggressive behavior by male quail have focused on ways to prevent the males from performing this agonistic behavior, either by beak trimming, anti-pecking devices, separation walls, removal of aggressive birds, reduced light intensity or simply reducing the number of males in the flock in relation to females, all with limited success. No attempts have been made to reduce the males’ motivation to perform this injurious behavior, for example, through selection. Quail selection, like chicken selection, has been focused mostly on production traits such as egg number and body weight (Minvielle, 1998; Cheng, 1992). This choice was appropriate for developing commercial lines quickly, but the genetics of other traits related to robustness (such as health and behavior) have been less investigated. With a potential transition on quail farms from cage to cage-free systems breeding quail which are robust and resilient to different environment, will be crucial to keep production sustainable, both from an animal welfare perspective as well as financially. In farm animal breeding, behavioral traits are rarely included in selection programs despite their potential to improve animal production and welfare by promoting animals that cope better in production systems (Goddard and Hayes, 2009; Meuwissen et al., 2016). In the past couple of decades, breeding goals have been broadened beyond production traits in many farm animal species to include health and functional traits (Olesen et al., 2000), such as calving ease and leg health in cattle (Egger-Danner et al., 2015) and broiler chickens (Kapell et al., 2012) and increased immunity in sheep (Silva et al., 2022) to name a few, and opportunities exists to increase the inclusion of behavior in breeding indices, as demonstrated for example by the identification of genes underlying anxiety in chickens (Johnsson et al., 2016; Wright and Henriksen, 2020). While the genetic diversity of farmed quail populations has rarely been studied, selection signatures and candidate genes associated with social behavior have recently been identified in the quail genome (Morris et al., 2020). Possibilities, therefore, exist to identify the genetic architecture underlying problematic behavior, such as aggression in Japanese quail, with the purpose of applying targeted genetic selection to reduced levels of, for example, aggressive behavior in males and thereby the occurrence of injuries on farms where males are part of the breeding and/or production (Frésard et al., 2012).

## Final words

In many parts of the world, Japanese quail meat and eggs are viewed as niche food, and quail production is still on a small scale compared to chickens. However, in terms of the number of individuals, Japanese quail production is comparable to other farmed animals. Moreover, the market has continued to grow in recent years without an equivalent increase in knowledge about how to best design their housing environment from an animal welfare perspective. While the development of sustainable cage-free housing for quail can lean on the large knowledge acquired from the chicken research on cage-free housing, it is also clear from the scientific literature covered in this review that their ecological background and behavioral needs differ quite a lot from those of chickens. Many knowledge gaps still exist when it comes to housing quail in any enclosure and would therefore greatly benefit from animal welfare-focused research regarding Japanese quail as production animals.

## Disclosures

The authors declare that this review was completed in the absence of any commercial or financial relationships that could be construed as a potential conflict of interest.
